# Farnesol as an antifungal agent: comparisons among *MTL* **a** and *MTLα* haploid and diploid *Candida albicans* and *Saccharomyces cerevisiae*

**DOI:** 10.3389/fphys.2023.1207567

**Published:** 2023-11-20

**Authors:** Cory H. T. Boone, Kory A. Parker, Daniel J. Gutzmann, Audrey L. Atkin, Kenneth W. Nickerson

**Affiliations:** School of Biological Sciences, University of Nebraska, Lincoln, NE, United States

**Keywords:** farnesol sensitivity, haploid specific genes, mating type, *Candida albicans*, *Saccharomyces cerevisiae*

## Abstract

**Aims:** Farnesol was identified 20 years ago in a search for *Candida albicans* quorum sensing molecules (QSM), but there is still uncertainty regarding many aspects of its mode of action including whether it employs farnesol transport mechanisms other than diffusion. Based on the structural similarity between farnesol and the farnesylated portion of the *MTL* **a** pheromone, we explored the effects of ploidy and mating type locus (*MTL*) on the antifungal activity of exogenous farnesol.

**Methods and results:** We approached this question by examining five *MTL* **a** and five *MTLα* haploid strains with regard to their farnesol sensitivity in comparison to six heterozygous *MTL* **a*/*** *α* diploids. We examined the haploid and diploid strains for percent cell death after exposure of exponentially growing cells to 0–200 µM farnesol. The heterozygous (*MTL* **a**/α) diploids were tolerant of exogenous farnesol whereas the *MTL* **a** and *MTL*α haploids were on average 2- and 4-times more sensitive, respectively. In the critical range from 10–40 µM farnesol their cell death values were in the ratio of 1:2:4. Very similar results were obtained with two matched sets of *MAT* **a**, *MAT*α, and *MAT* **a**/α *Saccharomyces cerevisiae* strains.

**Conclusion:** We propose that the observed *MTL* dependence of farnesol is based on differentially regulated mechanisms of entry and efflux which determine the actual cellular concentration of farnesol. The mechanisms by which pathogens such as *C. albicans* tolerate the otherwise lethal effects of farnesol embrace a wide range of physiological functions, including *MTL* type, ubiquinone type (UQ6-UQ9), energy availability, and aerobic/anaerobic status.

## 1 Introduction

The dimorphic fungus *C. albicans* is an important human pathogen. Yeast-hyphal interconversion is essential for its pathogenicity and in 2001 we identified the sesquiterpene E,E-farnesol as a quorum sensing molecule (QSM) able to block the yeast to hypha conversion in a cell density dependent manner (Hornby et al., 2001) and to inhibit biofilm formation (Ramage et al., 2002). Later we found that excreted farnesol also acted as a virulence factor for pathogenicity in mice (Navarathna et al., 2007; Hargarten et al., 2015) and it induced apoptosis in numerous fungi including *Aspergillus nidulans* and *Aspergillus fumigatus* (Semighini et al., 2006; Shirtliff et al., 2009). Thus, farnesol is a highly bioactive molecule (Nickerson et al., 2006). The potential role of farnesol and related molecules (i.e. 2, 3-dihydrofarnesol) as useful antifungal drugs has regained interest. For instance, Brasch et al. (2014) detailed their effectiveness versus dermatophytes while Katragkou et al. (2015) showed their synergistic effectiveness with fluconazole, amphotericin B, and micafungin versus *C. albicans* biofilms. More recently, Nagy et al. (2020) reported that farnesol inhibited growth and biofilm forming ability by *C. auris*, Dekkerova et al. (2022) confirmed a synergistic effect between farnesol and fluconazole in *C. auris*, and Nikoomanesh et al. (2023) confirmed a synergism between farnesol and either fluconazole or itraconazole which restored the original sensitivity of azole-resistant *C. albicans* and *Candida parapsilosis*. The idea that *C. albicans* secretes antifungal molecules as a mechanism of competition with other fungi is longstanding. Pure cultures of *C. albicans* were sometimes isolated from clinical lesions (Lewis and Hooper, 1943) while the dermatophyte *Trichophyton rubrum* did not grow when cocultivated with either *C. albicans* or spent medium from *C. albicans* (Jillson and Nickerson, 1948). The idea that this antifungal molecule was farnesol and that the target was actively respiring mitochondria was inherent in the observations of Machida and Tanaka (1999) and Machida et al. (1998), Machida et al. (1999). They showed a dose-dependent growth inhibition and ROS production for farnesol but not for other closely related molecules. Farnesol (3 isoprenes, C_15_) inhibited the growth of *S. cerevisiae* at 12–25 µM whereas geraniol (2 isoprenes, C_10_), geranylgeraniol (4 isoprenes, C_20_), farnesyl acetate, linalool, and squalene were not inhibitory at concentrations up to 200 or 400 µM. The mitochondrial involvement was clearly shown by the toxicity of 20–30 µM farnesol to *S*. *cerevisiae*, while petite mutants of *S. cerevisiae* (Machida et al., 1998; Fairn et al., 2007; Pathirana et al., 2020) and anaerobically grown *C. albicans* (Dumitru et al., 2004) were farnesol resistant.

An intriguing unanswered question concerns how 20–25 µM farnesol can kill or lyse potentially competing yeasts (Machida et al., 1998) and fungi (Semighini et al., 2006) as well as the opaque form of *C. albicans* (Dumitru et al., 2007) whereas the white form of *C. albicans* can tolerate 250–300 µM farnesol (Hornby et al., 2001; Ramage et al., 2002; Polke et al., 2017). What mechanisms does *C. albicans* use to protect itself from farnesol? This question applies to both exogenous farnesol and intracellular farnesol (Boone et al., 2022). We also know that the sensitivity of *C. albicans* to exogenous farnesol depends on the stage of growth at which the cells were stressed; cultures inoculated with stationary-phase cells tolerated up to 300 µM farnesol whereas those inoculated with exponential-phase cells were inhibited by 40 μM farnesol and killed by 100–300 µM farnesol (Uppuluri et al., 2007; Langford et al., 2010). At least three possible answers to this question have been tested. The first concerns the need for an energy source as was nicely shown by Shirtliff et al. (2009) wherein *C. albicans* cells underwent apoptosis after treatment with 40 µM farnesol if they had first been incubated for 24 h in phosphate-buffered saline (PBS). Both exponential- and stationary-phase cells were 2–10 times more likely to be lysed by farnesol when incubated in PBS than when incubated in growth media (Shirtliff et al., 2009; Langford et al., 2010). This need for an energy source to tolerate otherwise lethal detergent-like molecules duplicates our prior observation with *Enterobacter cloacae* growing in 1%–10% sodium dodecyl sulfate (SDS). Bacterial cells which entered stationary phase owing to carbon limitation were rapidly lysed by the SDS present whereas those that had entered owing to nitrogen or phosphorous limitation were not (Aspedon and Nickerson, 1993). An attractive model for how this energy dependence operates in *C. albicans* was presented by Liu et al. (2018) wherein farnesol activated the Tac1 and Znc1 transcription factors, thus inducing expression of the Cdr1, an ABC transporter and multidrug efflux pump, known to export phospholipids and possibly farnesol as well. The second idea concerns the types of ubiquinones synthesized by the microbes. Ubiquinones consist of a benzoquinone redox active ring system attached to a side chain with variable isoprenoid units. *S. cerevisiae* has six isoprenoid units in its side chain (UQ6), while most *Candida* sp. have seven (UQ7), and *C. albicans* and *C*. *dubliniensis* have nine (UQ9). Previously, we showed that *S. cerevisiae* engineered to produce UQ9 were ca. 5X less sensitive to exogenous farnesol than their parent *S. cerevisiae* and produced ca. 10X less ROS in response to a given level of farnesol (Pathirana et al., 2020). We then suggested that *C. albicans* and *C. dubliniensis* evolved to use UQ9 rather than UQ7 in part to protect themselves from their secreted farnesol (Pathirana et al., 2020). The third idea, and the one tested in this paper, is closely tied to the unanswered question of whether the lipophilic farnesol enters a cell via diffusion, facilitated diffusion, or active transport (Nickerson and Atkin, 2016). Both farnesol and the homoserine lactones used by Gram-negative bacteria were first identified following extraction of the active molecules into ethyl acetate (Eberhard et al., 1981; Hornby et al., 2001) and thus they both should have some capacity to enter via diffusion.

A variable capacity for diffusion was implicit in the studies of Polke et al. (2017) who identified *eed1∆/∆* as the first farnesol hypersensitive mutant of *C. albicans*. This mutant was 50-fold more sensitive to exogenous farnesol as a QSM and it also secreted ×10 more farnesol than its parent. Also, while able to form hyphae, *eed1∆/∆* could not maintain those hyphae (Polke et al., 2017). Then, in a MicroCommentary on the mechanistic implications of *eed1∆/∆*, we (Nickerson and Atkin, 2016) suggested that *C. albicans* has a regulated farnesol transporter which might be related to using a farnesylated peptide as a mating pheromone (Bennett et al., 2003; Dignard et al., 2007). The **a**-factor is a farnesylated peptide secreted by mating type **a** cells via an ABC transporter called Ste6p or Hst6p and then recognized and bound by mating type α cells via an **a**-factor receptor called Ste3p. Ste6p is in the plasma membrane of *MTL* **a** cells only and Ste3p is in the plasma membrane of *MTL*α cells only. We further suggested that Ste6p or Ste3p might transport free farnesol as well as the farnesylated peptide. A key point is that in MTL**a**/α heterozygotes, both **a**-specfic, α-specific, and haploid specific gene expression is turned off, at least in *S. cerevisiae*, suggesting that the evolutionary pressure for *C. albicans* to become diploid derived in part from its use of farnesol as a QSM and a virulence factor (Nickerson and Atkin, 2016); the secreted farnesol would otherwise have been toxic to one or both of the haploid cell types.

The present paper examines the farnesol sensitivity for six diploid and ten haploid strains of *C. albicans*. The haploid strains, five *MTL* **a** and five *MTL*α, were described by Hickman et al. (2013). Transport of exogenous farnesol by a haploid specific mechanism was corroborated in that the heterozygous diploid strains of *C. albicans* were 2.4 times more resistant to 20–40 µM farnesol than the *MAT* **a** haploid cells, and 4.6 times more resistant than the *MATα* haploid cells. Furthermore, the farnesol sensitivities of two *MTL*α/α and two *MTL* **a**/**a** cell lines matched those of the α haploid and **a** haploid cells, respectively. These results provide a partial explanation for why *C. albicans* is primarily diploid and why heterozygous diploid strains of *C. albicans* are more virulent than homozygous diploids (Lockhart et al., 2005).

## 2 Materials and methods

### 2.1 Yeasts, media, and culture methods

A complete list of the yeast strains is in Table 1. The haploid *C. albicans* cell lines were previously described and obtained from the Judith Berman laboratory (Hickman et al., 2013). The BY4741-BY4743 *S. cerevisiae* series was purchased from EUROSCARF, Oberusel, Germany. The W303 series was obtained from Susan Wente, Vanderbilt University, Nashville, Tennessee. Cultures were grown in YPD [1% (w/v) yeast extract, 2% peptone (w/v) and 2% dextrose (w/v)] media. A 3 mL overnight culture grown from a single colony was used to inoculate 50 mL YPD in 250 mL Erlenmeyer flask at an OD_600_ of 0.1. The 50 mL cultures were grown for 4–6 h at 30°C on a rotary shaker set at 270 RPM until they had reached ca. 2 × 10^7^ cells mL^−1^ (OD_600_ = 0.5) whereupon they were subdivided into six 25 mL Erlenmeyer flasks (5 mL) containing 0–200 µM farnesol in methanol and incubated as above for an additional 30 min. The negative control without farnesol received 50 µL methanol (1%), equivalent to the methanol added for 200 µM farnesol. The E, E-farnesol stock solution (Sigma product # 277541) was sealed under nitrogen and stored at −20°C. Note that farnesol has a maximum water solubility of only 1–1.2 mM (Knobloch et al., 1988).

**TABLE 1:** *Candida albicans* and *Saccharomyces cerevisiae* strains used in this study.

*Candida albicans*			
Strains	Ploidy	Genotype	Source
Heterozygous diploid			
SC5314	Diploid	WT *MTL**a***/α	Hornby et al. (2001)
A72	Diploid	WT	“
MEN	Diploid	WT	“
10261	Diploid	WT	“
3153A	Diploid	WT	“
RM1000	Diploid		Atkin/Nickerson Collection
*MTL**a*** haploid			
YJB12864	Haploid	*MTL**a***	Hickman et al. (2013)
YJB12814	Haploid	*MTL**a***	“
YJB12868	Haploid	*MTL**a***	“
YJB12870	Haploid	*MTL**a***	“
YJB12881	Haploid	*MTL**a***	“
*MTL*α haploid			
YJB12804	Haploid	*MTL*α	Hickman et al. (2013)
YJB12812	Haploid	*MTL*α	“
YJB12818	Haploid	*MTL*α	“
YJB12880	Haploid	*MTL*α	“
YJB12875	Haploid	*MTL*α	“
Homozygous diploid			
3294	Diploid	*MTL* ***a***/***a***	Dumitru et al. (2007)
3745	Diploid	*MTL* ***a***/***a***	“
3315	Diploid	*MTL*α/α	“
3740	Diploid	*MTL*α/α	“
*Saccharomyces cerevisiae*			
Strains	Ploidy	Genotype	Source
BY4741	Haploid	*MAT**a***	Brachmann et al. (1998)
BY4742	Haploid	*MAT*α	“
BY4743	Diploid	*MAT**a***/α	“
W303-1A	Haploid	*MAT**a***	Wente and Blobel (1993)
W303-1B	Haploid	*MAT*α	“
W303	Diploid	*MAT**a***/α	“

### 2.2 Cell vitality by flow cytometry

**FIGURE 1:**
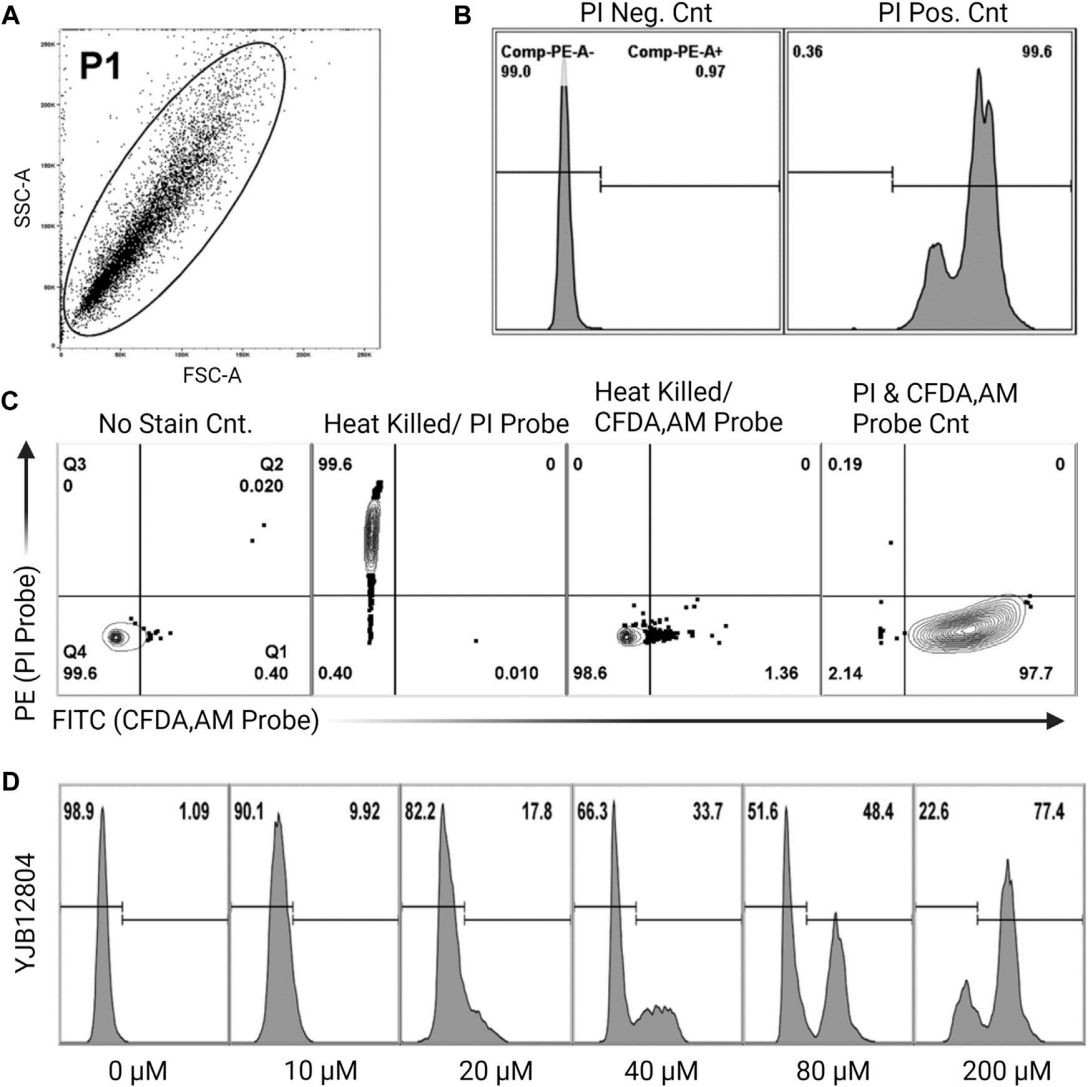
Experimental setup for percent cell viability using flow cytometry **(A)**. Representative side scatter/forward scatter (SSC/FSC) plot gated to include whole cells (the P1 population) but exclude sample debris from analysis **(B)**. Cell death histogram for a non-stained sample (PI negative control) and heat killed sample (PI positive control) **(C)**. Four controls showing how the gates were defined. Live cells, no stain control; heat killed cells, single PI probe; heat killed cells, single CFDA-AM probe; live cells, double probe. For PI staining, the gate is the horizontal line, cells above the line are dead. For CFDA-AM staining, four vertical lines are the gates, cells to the right of the line are viable. These controls determine the correct number of cells to use, to avoid having non-stained cells biasing the results **(D)**. Sample histograms show the progression from live to dead cells following treatment with increasing concentrations of farnesol.

**FIGURE 2:**
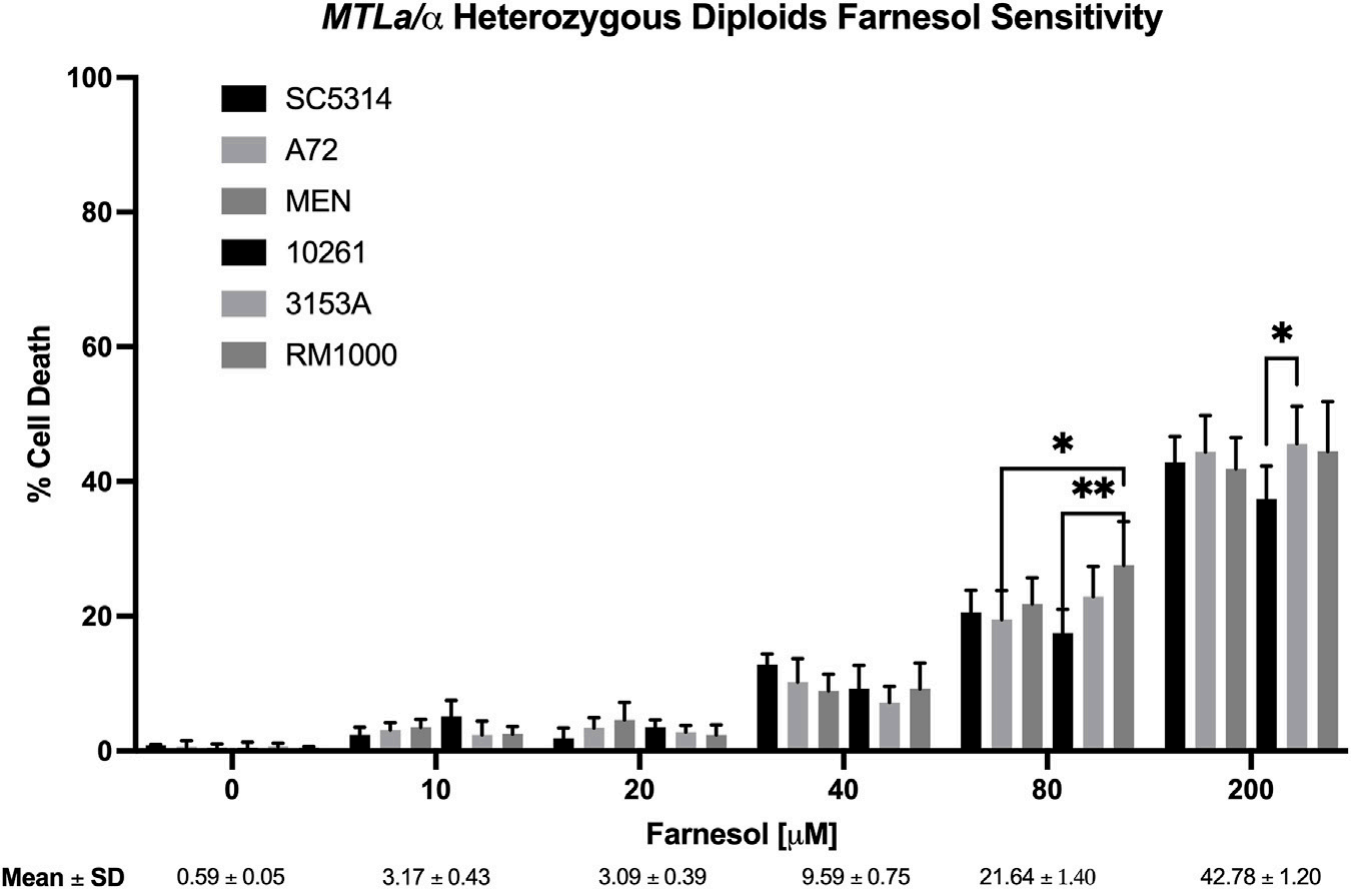
*Candida albicans* wild-type diploid farnesol sensitivity at increasing farnesol concentrations. Mean ± SEM across strains were calculated for strains SC5314, A72, MEN, 10261, and 3153A only. RM 1000 is an auxotrophic mutant derived from SC5314. Statistical analysis by 2-way ANOVA with Tukey’s *post hoc* correction. *p < 0.05, **p < 0.01.

**FIGURE 3:**
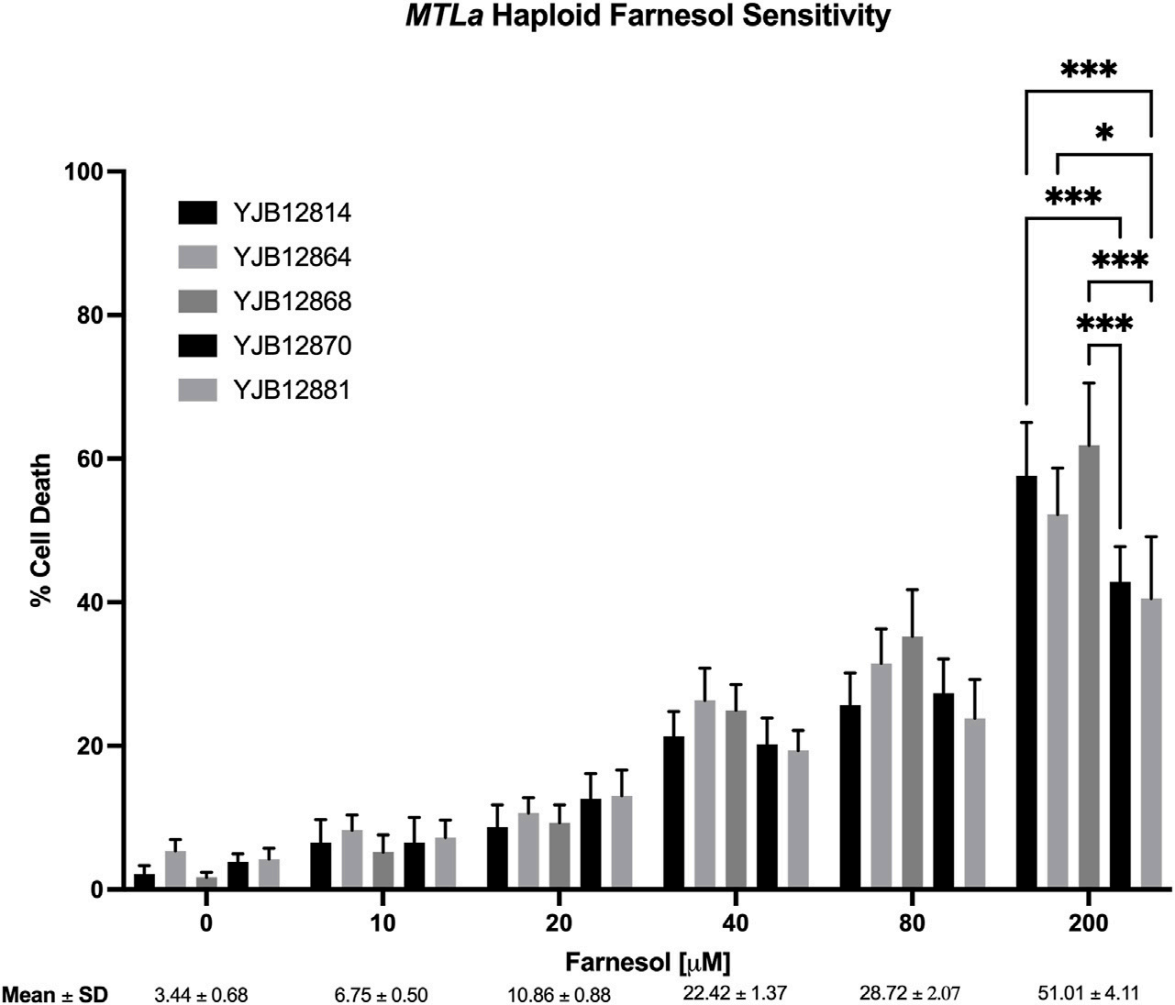
*Candida albicans MTL* **a** farnesol sensitivity at increasing farnesol concentrations. Mean ± SEM across strains were calculated. For each farnesol concentration, the cluster of 5 bars reflect YJB 12814, 12864, 12868, 12870, and 12881 reading from left to right. Statistical analysis by 2-way ANOVA with Tukey’s *post hoc* correction. *p < 0.05, **p < 0.01, ***p < 0.001.

**FIGURE 4:**
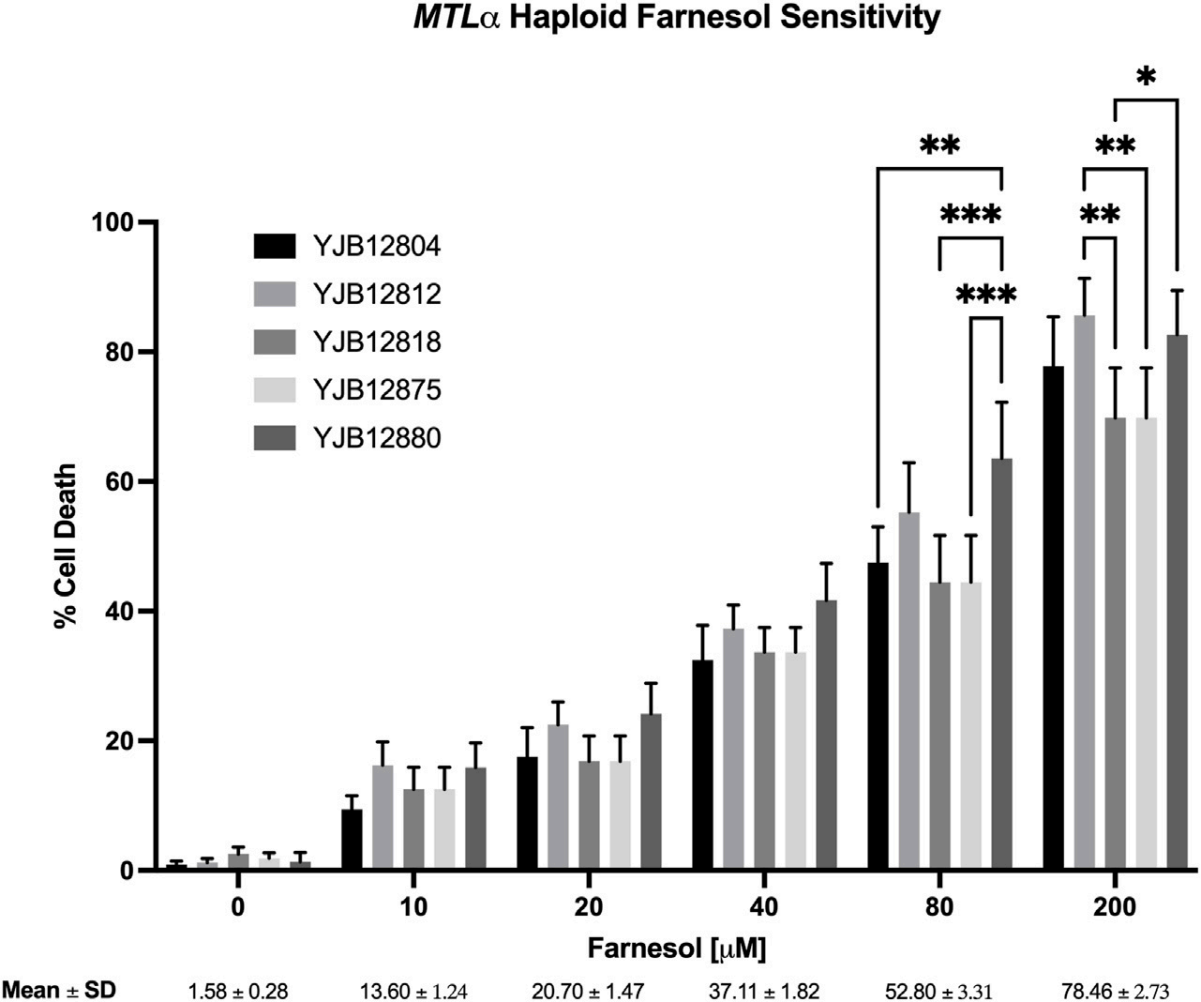
*Candida albicans MTLα* farnesol sensitivity at increasing farnesol concentrations. Mean ± SEM across strains were calculated. For each farnesol concentration, the cluster of 5 bars reflect YJB 12804, 12812, 12818, 12875, and 12880 reading from left to right. Statistical analysis by 2-way ANOVA with Tukey’s *post hoc* correction. *p < 0.05, **p < 0.01, ***p < 0.001.

**FIGURE 5:**
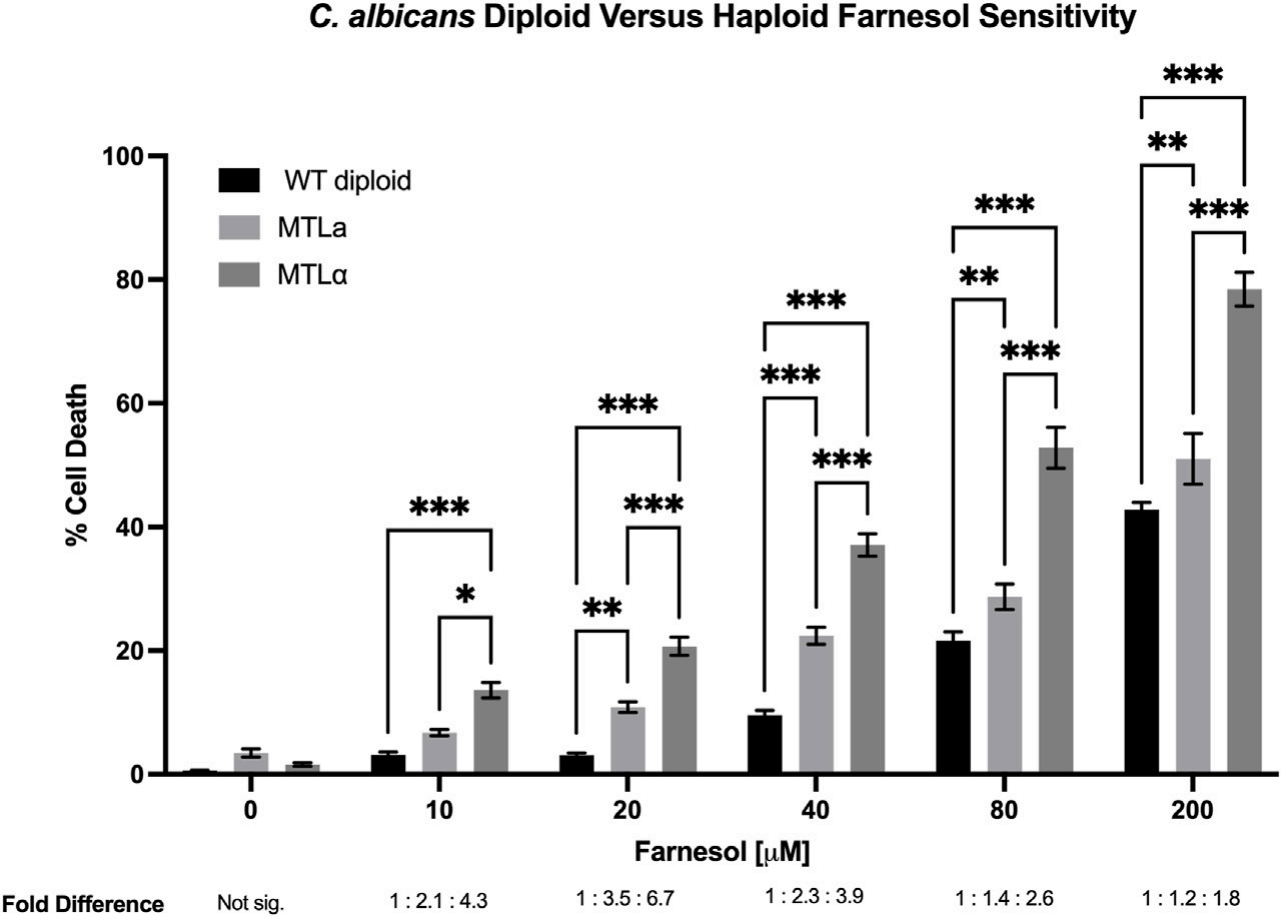
*Candida albicans* wild-type diploid versus *MTL* **a** versus *MTLα* haploid farnesol sensitivity at increasing farnesol concentrations. Mean ± SEM signified by errors bars are from Figures 3, 4, this figure, respectively. The average fold differences are reported below [F], with the diploid averages assigned a value of 1. At 0 μM farnesol fold difference is not significant because total cell death was miniscule. Statistical analysis by 2-way ANOVA with Tukey’s *post hoc* correction. *p < 0.05, **p < 0.01, ***p < 0.001.

**FIGURE 6:**
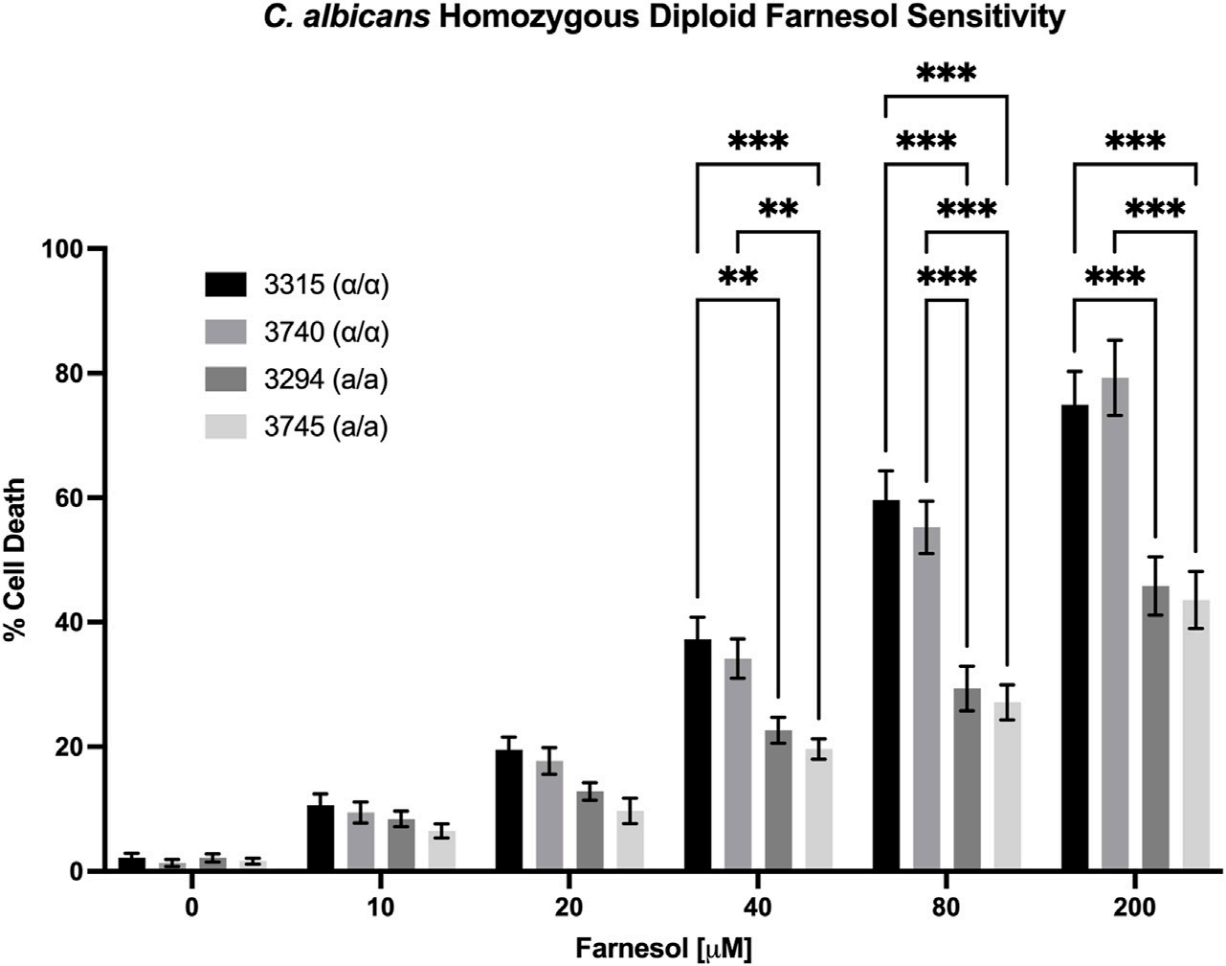
*Candida albicans* homozygous diploid farnesol sensitivity. Strains 3,315 and 3,740 = *MTLα/α*; strains 3,294 and 3,745 = *MTL* **a** */* **a**. Statistical analysis by 2-way ANOVA with Tukey’s *post hoc* correction. *p < 0.05, **p < 0.01, ***p < 0.001.

Cell vitality was assessed as shown in Figures 1A–D using FungaLight Yeast Vitality Kit (Life Technologies) combined with FACS (Boone et al., 2017). The kit contains the cell permeable, non-specific esterase substrate 5-carboxyfluorescein diacetate, acetoxymethyl ester (CFDA,AM) to assess esterase housekeeping protein activity, and the cell impermeable, DNA intercalating agent, Propidium Iodide (PI) to assess membrane integrity. Probe fluorescence indicative of cell death of individual cells to varying concentrations of farnesol exposure was measured on a BDFACS Canto II (BD Biosciences, San Jose CA, United States) instrument interfaced with FACS Diva v6.11 software (Becton, Dickinson and Co., Franklin Lakes NJ, United States) and analyzed using Flowjo v10.2 software (TreeStar Inc., Ashland OR, United States). Instrument setup, acquisition, and data analysis using non-stained and single-stained controls was performed as suggested by ISAC, the International Society for Analytical Cytometry (Lee et al., 2008). Following the 30-min incubation with farnesol, 1 mL aliquots were harvested by centrifugation (10,000 g at 4°C), washed once with an equal volume of PBS, diluted 10-fold (to 10^6^ cells mL^−1^) in PBS with 0.01% Tween 20, and incubated with 2 µM CFDA, AM and 9 µM PI probes for 20 min in the dark, and analyzed by FACS. Non-stained, single probe stained, and double probe stained controls were used to ensure that a correct number of cells (10^6^ cells mL^−1^) were used to prevent non-stained cells biasing results (non-stained population <5%). PE channel (PI stained or non-stained populations) histograms were used to assess the percentage of dead cells in the cultures, see Figure 1D. Thus, each run generates a live/dead histogram and the values reported in Figures 2–6 are the mean ± SEM of three histograms.

### 2.3 *Saccharomyces* farnesol stress--cell counting following PI staining

**FIGURE 7:**
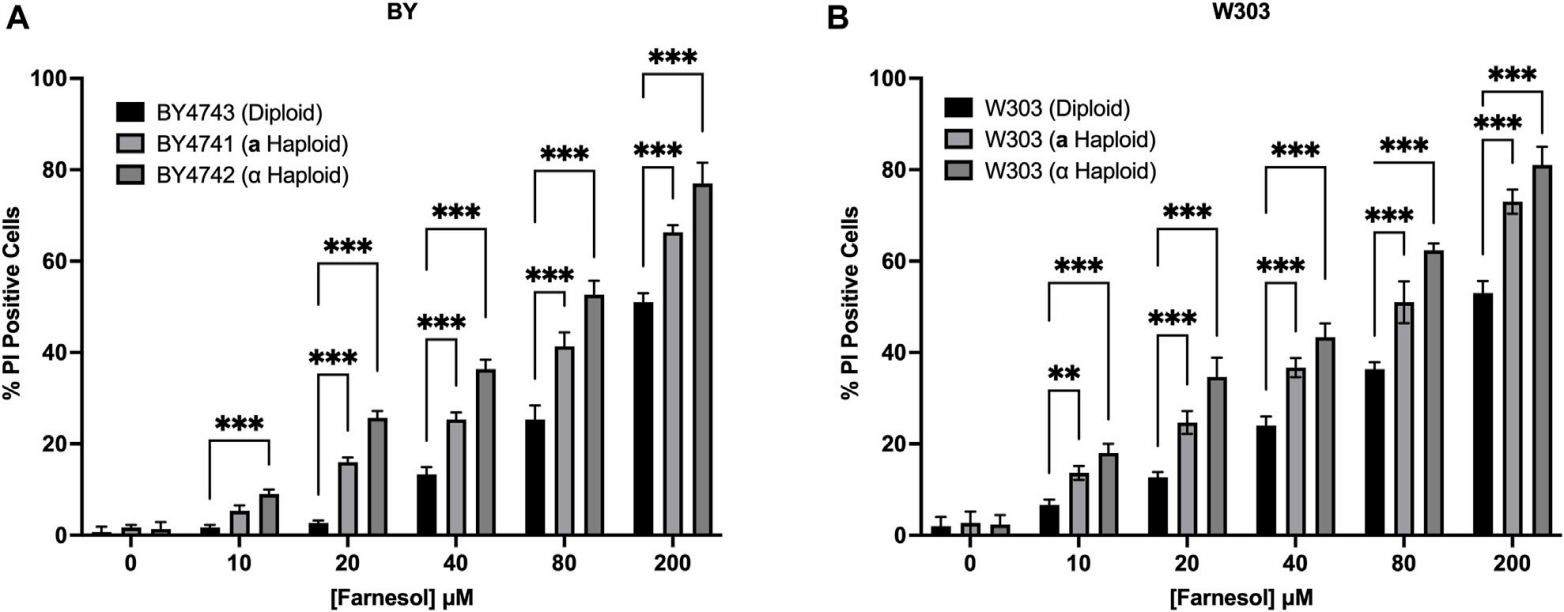
*Saccharomyces cerevisiae* diploid versus *MAT* **a** versus *MATα* haploid farnesol sensitivity at increasing farnesol concentrations **(A)**. BY4741-4743 series **(B)**. W303 series. Mean ± SD (*n* = 3) with statistical analysis by 2-way ANOVA with Tukey’s *post hoc* correction. **p < 0.01, ***p < 0.001.

*S. cerevisiae* cells were grown at 30°C in 500 mL flasks with 100 mL liquid GPP (Hornby et al., 2001). To fulfill their respective auxotrophies, the BY471, BY4742, and BY4743 strains had added Leu, Lys, Met, His, and uracil and the W303 series had added Trp, Leu, His and adenine (all at 40 μg/mL). After ca. 10 h growth the mid-log cultures (OD_600_ = 2.0–3.5) were diluted with fresh media to OD_600_ = 2.0 and then subdivided to compare their farnesol tolerance. For each strain, 10 mL of culture was aliquoted into each of a series of 50 mL flasks containing 0, 10, 20, 40, 80, and 200 µM farnesol and shaken at 220 RPM for 1 h at 30°C. Then 500 µL of the farnesol-stressed culture was mixed with 500 µL of propidium iodide (12 μg/mL) for a final PI concentration of 6 μg/mL and incubated for 10 min in the dark (Davey and Hexley, 2011). The PI stock (MP Biochemicals, Solon, Ohio) was 3 mg/mL in methanol, subsequently diluted by 40 µL in 10 mL water (12 μg/mL). In no case did the methanol exceed 1%. After their incubation with PI, the cultures were diluted 1:10 in water so that their cell densities better fit the dynamic range of the Countess II FL automated cell counter (Life Technologies, Carlsbad, CA). Ten µL of culture was loaded into each chamber of the dual chamber cell counting slides (Invitrogen), thus providing technical duplicate values. Bright field intensity was set at 30 and red fluorescence (RFP) at 61. These values were chosen based on control cultures with less than 5% PI positive cells. Small debris was size excluded prior to analysis. Dead cells exhibited red fluorescence. Percent dead cells was measured in technical duplicate and biological triplicate. Data are biological triplicate means ± SD (Figure 7) with live/dead ratios based on the percent of total cells which are positive (red); they are not dependent on cell size or the PI intensity per cell.

### 2.4 Statistical analysis

Statistical analyses were performed using Microsoft Excel (Version 16.52) and GraphPad Prism Software (Version 9.1.2). All data are represented as mean ± SEM unless otherwise stated. Normal distribution was accessed by visual inspection of Q-Q plots. Differences between two or more groups were accessed by Two-way ANOVA with Tukey’s *post hoc* test if differences were significant. Differences were considered significant at *p* < 0.05. Significance is denoted as *p < 0.05, **p < 0.01, ***p < 0.001.

## 3 Results

### 3.1 Farnesol sensitivity of wild type diploid *C. albicans*

Previous studies on the toxicity of exogenous farnesol to fungi have ignored the question of how farnesol enters the responding cell (Machida et al., 1998; Semighini et al., 2006). One explanation for why clinical isolates of *C. albicans* are so tolerant of exogenous farnesol invokes entry assisted by transport systems active in haploid cells but turned off or minimized in *MTL* **a**/α heterozygous diploid cells. Step one in testing this hypothesis is establishing a baseline for diploid *C. albicans*, i.e., not just strain SC5314.

Figure 2 shows the % cell death values for six diploid strains of *C. albicans* which had been stressed with 0–200 µM farnesol, strains SC5314, A72, MEN, 10261, 3153A, and RM1000. The negative control (0 µM farnesol) showed that 1% methanol was not harmful to any of the strains tested. The percent cell death values were very similar for all strains; they were effectively resistant to ≤20 µM exogenous farnesol and then exhibited 10, 20, and 44% cell death at 40, 80, and 200 µM farnesol, respectively (Figure 2). The appearance of significant cell death at 80 and 200 µM farnesol (Figure 2) does not contradict prior statements that *C. albicans* is resistant to 250–300 µM farnesol (Hornby et al., 2001; Ramage et al., 2002) because of differences in the experimental design. In Figure 2 farnesol was added to actively growing, exponential phase cells, which are more sensitive to farnesol than nongrowing cells exposed to farnesol from time zero (Uppuluri et al., 2007; Langford et al., 2010). For strain SC5314, the % death values with increasing farnesol were confirmed by CFU values on YPD plates (data not shown) and all subsequent experiments (Figures 2–4, 6) included SC5314 as a positive control.

### 3.2 Haploid *C. albicans* are more sensitive to exogenous farnesol than are diploid strains

Next, ten haploid strains, five *MTL* **a** (Figure 3) and five *MTLα* (Figure 4), were analyzed by FACS for their farnesol sensitivities, whereupon their average % cell death values were compared to those previously found for the diploids (Figure 5). The *MTL* **a** (Figure 3) and *MTLα* (Figure 4) haploids gave tight clusters with low standard deviation; they exhibited significant cell death even at 10 µM farnesol, the lowest concentration tested. For farnesol concentrations from 10 to 80 μM, the average % cell death values for the α haploids were roughly 2-times that for the **a** haploids (Figure 5) and for all concentrations, the cell death values were in the order: diploid < **a** haploid < α haploid (Figure 5). For the concentrations which we deem most physiologically significant (20–40 µM), the relative cell death values were ca. 1:2:4 (Figure 5).

### 3.3 The farnesol sensitivity of homozygous diploids reflects their *MTL* genotype, not their ploidy

We also examined the farnesol sensitivity of four *MTL* homozygous diploids previously used to demonstrate anaerobic mating in *C. albicans* (Dumitru et al., 2007). As seen in Figure 6, the homozygous diploids exhibited % cell death values comparable with those observed for the corresponding haploid strains. The two *MTLα/α* strains (3,315 and 3,740) matched the high cell death rates of the *MTLα* haploids (Figure 4) while the two *MTL* **a** */* **a** strains (3,294 and 3,745) matched those of the *MTL* **a** haploids (Figure 3). These data suggest that it is the combination of *MTL* **a** */α* in the heterozygous diploids which confers farnesol resistance rather than the duplicated DNA content.

### 3.4 Haploid *S. cerevisiae* are more sensitive to exogenous farnesol than are diploid strains

The rationale for why diploid cells of *C. albicans* might be more farnesol tolerant than haploid cells applies equally to the model yeast *S. cerevisiae* because it too employs a farnesylated peptide as the **a**-pheromone (Michaelis and Barrowman, 2012). Two matched sets of *S. cerevisiae* diploid/**a** haploid/α haploid strains were examined in a PI based live/dead assay (Figure 7). All the negative control cultures (without added farnesol) had <5% PI positive cells. The results for the BY (Figure 7A) and W303 (Figure 7B) series were almost identical. As expected, the % PI positive cells were 20%–30% greater for *S. cerevisiae* (Figure 7) than for *C. albicans* (Figure 5) and the cell death values for all farnesol concentrations were in the same order: diploid < **a** haploid < α haploid (Figure 7). We conclude that the underlying mechanisms for the greater farnesol resistance exhibited by diploid yeasts are likely the same.

## 4 Discussion

The adaptations made by a potential pathogen to become a successful pathogen are important and intriguing. *C. albicans* employs farnesol as one of its several virulence factors (Navarathna et al., 2007; Hargarten et al., 2015) and farnesol is a highly bioactive molecule. It is toxic to many fungi (Semighini et al., 2006) and its mode of action is thought to include interaction with fungal mitochondria leading to lethal levels of ROS production (Machida et al., 1998) and apoptosis (Semighini et al., 2006; Shirtliff et al., 2009). Any organism producing an antibiotic or other bioactive, biocidal molecule must perforce evolve mechanisms to protect itself from that molecule. We previously suggested that *C. albicans* and *C. dubliniensis* evolved to use UQ9 rather than UQ7 in part to protect themselves from their secreted farnesol (Pathirana et al., 2020). We now suggest that the push towards diploidization may have served a similar purpose. We have taken advantage of the collection of **a** and α haploid isolates of *C. albicans* (Hickman et al., 2013) to examine their sensitivity to exogenous farnesol. Marked differences were observed. With regard to farnesol sensitivity, all strains showed increasing percent cell death by flow cytometry as the farnesol concentration increased from 0 to 200 µM. However, the farnesol cell death values for the **a**/α diploid strains were always far less than for the haploid strains, with the % cell death values for diploids, **a** haploids, and α haploids being in the ratio of 1:2:4 (Figure 5). Thus, the higher farnesol sensitivity of α cells presents the opportunity for one haploid cell type to kill the other. These data are consistent with the suggestion that the secretion of farnesol as a QSM and as a virulence factor provided an evolutionary driving force for the diploidization of *C. albicans* (Nickerson and Atkin, 2016).

These results show the correlation of mating type with farnesol sensitivity (entry) and they are consistent with most of the predictions made by Nickerson and Atkin (2016). These predictions were based on both *S. cerevisiae* (Michaelis and Barrowman, 2012) and *C. albicans* (Dignard et al., 2007) using farnesylated peptide pheromones secreted by *MTL* **a** strains and then bound by *MTLα* strains. These functions are accomplished by Ste6p in *MTL* **a** and Ste3p in *MTLα*, respectively. Because both of these proteins are able to distinguish the farnesylated and non-farnesylated peptides, we hypothesized that they or related proteins would also be able to transport farnesol, or allow passage of farnesol, possibly at a reduced efficiency. The data presented in this paper are consistent with the idea that the haploid strains are more sensitive to exogenous farnesol because they have and express a set of genes that are unique to the *MTL* **a** and *MTL*α strains, or any other gene which is transcribed in a ploidy-specific or mating type specific manner.

As an intriguing possibility, Srikantha et al. (2012) showed that the *MTL* locus of *C. albicans* contained the mating type genes and also three apparently unrelated “nonsex” genes, the essential phosphatidyl inositol kinase gene, *PIK*, the poly(A) polymerase gene, *PAP*, and the nonessential oxysterol binding protein gene, *OBP*. The *MTL* **a** and *MTL*α loci both contain all three nonsex genes. Importantly, the DNA homologues of the **a** and α copies of *PIK*, *PAP*, and *OBP* were 58, 66, and 66% while the identity of the deduced **a** and α proteins encoded by *PIK*, *PAP*, and *OBP* were 81, 89, and 91%, respectively (Srikantha et al., 2012). These numbers are in marked contrast to the DNA homology (99.5%) and protein identity (99.6%) values for 50 genes neighboring the *MTL* locus (Srikantha et al., 2012).

The potential importance of these nonsex genes is evident in prior work from the Soll laboratory (Yi et al., 2011) when they showed that the biofilms produced by *C. albicans MTL* **a**/α cells differed dramatically from those produced by *MTL* **a**/**a** or *MTL*α/α cells. The biofilms were similar morphologically, but they were structurally and functionally distinct. In particular, biofilms formed by *MTL* **a**/α cells were impermeable to molecules in the size range of 140–300 Da and resistant to many antifungals whereas the *MTL* **a**/**a** and *MTL*α/α biofilms were permeable to molecules in this size range and susceptible to those antifungals (Yi et al., 2011). Farnesol has a molecular weight of 222, Srikantha et al. (2012) showed that *OBP* was an essential gene for biofilm impermeability and fluconazole resistance. Since the biofilms are morphologically indistinguishable, this difference in susceptibility suggests critical differences at the individual cell level.

Currently, our data show the importance of ploidy and mating type (**a** vs. α); they do not show anything further regarding an actual mechanism. Another possibility is that the ploidy state and/or mating type may determine more or less active farnesol metabolism in the cell, hence more or less resistance to growth inhibition or killing by farnesol. In this regard, neither *C. albicans* nor *S. cerevisiae* appears to have the two-step farnesol salvage pathway (Crick et al., 1997) whereby farnesol is converted to FPP (Verdaguer et al., 2022). This pathway is present in plants, animals, and bacteria but the relevant genes (*VTE5* and *VTE6* from *Arabidopsis thaliana*) have no homologs in either the *Candida* or *Saccharomyces* Genome Databases. Other regulated mechanisms for farnesol metabolism are still possible. In Boone et al. (2022) we followed the intracellular farnesol levels (F_i_) for *C. albicans* SC5314 over 3 days of growth. The per cell F_i_ increased continuously during exponential growth but then decreased during stationary phase until they returned to their pre-growth levels. Such a decrease in F_i_ could have many explanations. However, a subsequent study of 164 transcription regulator knockout mutants in the Homann collection (Homann et al., 2009) identified two high-accumulating mutants that did not exhibit the decay in farnesol levels during stationary phase, suggesting that an otherwise uncharacterized farnesol modification/degradation mechanism is absent in these mutants (Gutzmann et al., 2023). Clearly there is an abundance of possible mechanisms for the ploidy and mating type specific differences we have observed.

## Data Availability

The original contributions presented in the study are included in the article/Supplementary material, further inquiries can be directed to the corresponding author.
